# Obesity and Hepatic Steatosis Are Associated with Elevated Serum Amyloid Beta in Metabolically Stressed APPswe/PS1dE9 Mice

**DOI:** 10.1371/journal.pone.0134531

**Published:** 2015-08-05

**Authors:** Feng-Shiun Shie, Young-Ji Shiao, Chih-Wen Yeh, Chien-Hung Lin, Tsai-Teng Tzeng, Hao-Chieh Hsu, Fong-Lee Huang, Huey-Jen Tsay, Hui-Kang Liu

**Affiliations:** 1 Center for Neuropsychiatric Research, National Health Research Institutes, Zhunan, Taiwan, ROC; 2 Division of Basic Chinese Medicine, National Research Institute of Chinese Medicine, Taipei, Taiwan, ROC; 3 Institute of Biopharmaceutical Science, National Yang-Ming University, Taipei, Taiwan, ROC; 4 Institute of Neuroscience, Brain Research Center, National Yang-Ming University, Taipei, Taiwan, ROC; 5 Institute of Anatomy and Cell Biology, National Yang-Ming University, Taipei, Taiwan, ROC; 6 Ph.D. Program for Clinical Drug Discovery from Botanical Herbs, Taipei Medical University, Taipei, Taiwan, ROC; University of Florida, UNITED STATES

## Abstract

Diabesity-associated metabolic stresses modulate the development of Alzheimer’s disease (AD). For further insights into the underlying mechanisms, we examine whether the genetic background of APPswe/PS1dE9 at the prodromal stage of AD affects peripheral metabolism in the context of diabesity. We characterized APPswe/PS1dE9 transgenic mice treated with a combination of high-fat diet with streptozotocin (HFSTZ) in the early stage of AD. HFSTZ-treated APPswe/PS1dE9 transgenic mice exhibited worse metabolic stresses related to diabesity, while serum β-amyloid levels were elevated and hepatic steatosis became apparent. Importantly, two-way analysis of variance shows a significant interaction between HFSTZ and genetic background of AD, indicating that APPswe/PS1dE9 transgenic mice are more vulnerable to HFSTZ treatment. In addition, body weight gain, high hepatic triglyceride, and hyperglycemia were positively associated with serum β-amyloid, as validated by Pearson’s correlation analysis. Our data suggests that the interplay between genetic background of AD and HFSTZ-induced metabolic stresses contributes to the development of obesity and hepatic steatosis. Alleviating metabolic stresses including dysglycemia, obesity, and hepatic steatosis could be critical to prevent peripheral β-amyloid accumulation at the early stage of AD.

## Introduction

Alzheimer’s disease (AD) involves aberrant protein processing and is characterized by excessive accumulation of β-amyloid (Aβ), which is derived from cleavage of the amyloid precursor protein (APP) [1]. Approximately 99% of AD patients have the sporadic form of the disease (with no known genetic basis). The environmental risk factors for sporadic AD are believed to include insulin resistance-related diseases, such as type 2 diabetes mellitus, obesity, non-alcoholic fatty liver disease, and metabolic syndrome [2–6].

Intact insulin and insulin-like growth factor 1 (IGF-1) signal transduction in the central nervous system (CNS) may preserve normal brain structure and function by attenuating tau protein hyperphosphorylation, Aβ accumulation, and neuronal death [7]. On the other hand, systemic insulin signaling plays a major role in nutrient metabolism. However, the role of systemic insulin resistance in AD pathogenesis remains controversial and is currently under intensive investigation [8, 9].

By studying transgenic mice, acceleration of AD pathogenesis in model systems has been achieved by introducing systemic insulin resistance conditions, including dietary manipulation (e.g., high-fat diet [HFD]), leptin knockout (ob/ob), mutant leptin receptor (db/db), and nitrosamine exposure (i.e., administration of streptozotocin [STZ]) [10–13]. AD mice subjected to these additional manipulations showed increased Aβ plaque burdens, tau protein hyperphosphorylation, cerebrovascular inflammation, and structural alterations in the brain.

Interestingly, accumulating evidence from investigations of the etiological roles of systemic diseases in AD mouse models has revealed an overlooked situation: that AD genetic background may also conversely affect systemic insulin sensitivity and metabolism [14]. First, when fed an HFD, the weight gain and glucose intolerance in APP/PS1^(A246E)^ double-transgenic mice became more severe than in wild-type mice and PS1^(A246E)^ single-transgenic mice [15]. Second, when APP^+^-ob/ob mice were generated by crossing ob/ob (leptin homozygous mutants) mice with APP23 transgenic mice [11], further increases in hyperglycemia, hyperinsulinemia, and insulin resistance were observed. Furthermore, APP^+^-ob/ob and APP/PS1-db/+ mice have shown increased glucose intolerance and insulin resistance compared with ob/ob mice [11, 12]. And third, increased plasma Aβ levels have been found in AD patients with hyperglycemia, and Aβ directly induced hepatic insulin resistance [16, 17]. Intracerebral ventricle injection of soluble Aβ induces peripheral glucose intolerance and insulin resistance in muscle [18]. Therefore, current research evidence indicates that metabolic dysfunction and AD genetic background may interact reciprocally to exacerbate systemic metabolism and AD pathologies.

Among the current paradigms to induce metabolic disorders, the model combining dietary manipulation (HFD) and nitrosamine exposure (STZ) can cause most of the symptoms related to diabetes mellitus, including hyperglycemia, obesity, insulin resistance, glucose intolerance, and hepatic steatosis. In addition, this HFSTZ model is responsive to major anti-diabetic medications, such as glipizide and pioglitazone [19, 20]. Therefore, this inducible model reflects diabetes mellitus-related metabolic stresses.

As many cellular and behavioral abnormalities presumably occur far in advance of the appearance of AD pathology, elucidating the regulatory mechanisms of peripheral metabolic stresses in the prodromal stage of AD is critical for the development of effective AD therapies. To assess the impact of AD’s genetic background on metabolic index at the onset of AD pathogenesis, we comprehensively examined the metabolic characteristics of young adult APPswe/PS1dE9 transgenic mice subjected to HFSTZ treatment.

## Methods

### Animal handling

Male APPswe/PS1dE9 transgenic mice (No. 005864) were purchased from Jackson Laboratory (Bar Harbor, ME, USA) to breed with female wildtype C57BL/6J mice. Animals were housed under controlled room temperature (24 ± 1°C) and humidity (55–65%) with a 12:12-h (07:00–19:00) light-dark cycle. Experiments were conducted using male C57BL/6J wild-type (WT) siblings and APPswe/PS1dE9 transgenic (AD) mice. The present investigation was approved by the Animal Research Committee at the National Yang-Ming University (IACUC 1021271).

### Inducing metabolic stress with HFSTZ

HFSTZ has been shown to induce metabolic stresses such as hyperglycemia, obesity, insulin resistance, and glucose intolerance [19–21]. Male C57BL/6J wild-type (WT) siblings and APPswe/PS1dE9 transgenic (AD) mice were fed with a normal chow diet (NCD, MF-18, Oriental Yeast Co. Ltd., Tokyo, Japan) with water ad libitum. At the age of 10 weeks, half of WT and AD mice randomly chosen were fed with an HFD (60% energy from fat, TestDiet, St. Louis, MO, USA). After 2 weeks, HFD-fed mice were intraperitoneally injected with 50 mg/kg STZ as the HFSTZ induction group. NCD-fed mice were injected with vehicle (0.1 M citrate buffer, pH 4.5). Four experimental groups including NCD WT, NCD AD, HFSTZ WT, and HFSTZ AD mice were sacrificed after 11 weeks of dietary manipulations. The average weight of NCD WT, NCD AD, HFSTZ WT, and HFSTZ AD mice prior to the dietary manipulations were not significantly different (24.70 ± 0.69, 23.85 ± 0.93, 26.07 ± 0.65, and 25.73 ± 0.62, respectively).

### Oral glucose tolerance test

Mice were fasted for 16 h before oral glucose tolerance tests (OGTTs) after 6 weeks of dietary manipulations. Glucose solution (3 g/kg; Sigma Aldrich, St. Louis, MO, USA) was administered to WT and AD mice by oral gavage. Blood glucose was measured using a glucometer (Bioptik Technology, Taipei, Taiwan).

#### Serum analysis

Mice were injected with 80 mg/kg sodium pentobarbital intraperitoneally for deep anesthesia perfused with 50 ml saline and blood samples were collected by the cardiac puncture after 11 weeks of dietary manipulations. Serum triglyceride (TG), glutamate oxaloacetate transaminase (GOT), and glutamic-pyruvic transaminase (GPT) were measured by FUJI DRI-CHEM 3000 analyzer (Fujifilm, Tokyo, Japan). In addition, HDL, LDL, and VLDL cholesterol were measured by commercial kits (Abcam, Cambridge, UK). Furthermore, free fatty acids and leptin were measured by commercial kits purchased from BioVision (Miltipas, CA, USA) and R&D Systems (Minneapolis, MN, USA) after 11 weeks of dietary manipulation. Plates were read at a wavelength of 450 nm using the TECAN GENios plate reader and results were analyzed with Magellan version 7.0 software.

Serum insulin levels were determined with an Insulin-Kit HTRF (Cisbio, Codolet, France). The fluorescence intensities were measured on a SpectraMax M5 microplate reader (Molecular Devices, Sunnyvale, CA, USA). HOMA-IR (fasting blood glucose [mM] × fasting insulin [U/mL]/22.5) scores were then calculated.

### Measurement of hepatic triglyceride

Liver samples were homogenized in 100 mg/mL water containing 5% NP-40 (Sigma Aldrich); and TG in samples was assessed as recommended by the manufacturer (BioVision, Milpitas, CA, USA). Plates were read at a wavelength of 570 nm using the TECAN GENios plate reader.

### Aβ measurement by ELISA

The serum levels of Aβ40 and Aβ42 were measured with an Aβ ELISA kit purchased from Invitrogen (Carlsbad, CA, USA). Plates were read at a wavelength of 450 nm using the TECAN GENios plate reader.

The densitometric values were normalized to the total protein concentration.

### Histological analysis

Liver and epididymal fat tissues were postfixed in 4% formaldehyde at 4°C overnight. Epididymal fat tissues were paraffin embedded. 5-μm-thick fat tissue sections were stained with hematoxylin and eosin (HE). 30-μm-thick frozen liver sections were stained with Oil Red O. The images of HE- and Oil Red O-stained sections in four random fields were taken under a light field microscope (BX63, Olympus, Tokyo, Japan). Adipocyte size was measured using ImageJ software (National Institutes of Health, Bethesda, MD, USA).

### Statistical Analysis

Statistics were performed using GraphPad Prism (GraphPad, La Jolla, CA, USA) and SPSS (IBM, Armonk, NY, USA) software. All values are given as mean ± standard error of the mean (SEM). Data was analyzed by one-way analysis of variance (ANOVA) followed by Tukey’s honest significant difference (HSD) post hoc test. Comparisons of serum Aβ were performed using unpaired Student’s t-tests. The interaction and effect between factors were analyzed by two-way ANOVA (general linear model). For all tests, *p* < 0.05 was considered significant. The correlation between serum Aβ and metabolic markers was assessed with Pearson’s correlation analysis.

## Results

### HFSTZ-mediated metabolic stress was exacerbated in APPswe/PS1dE9 mice

**Fig 1:**
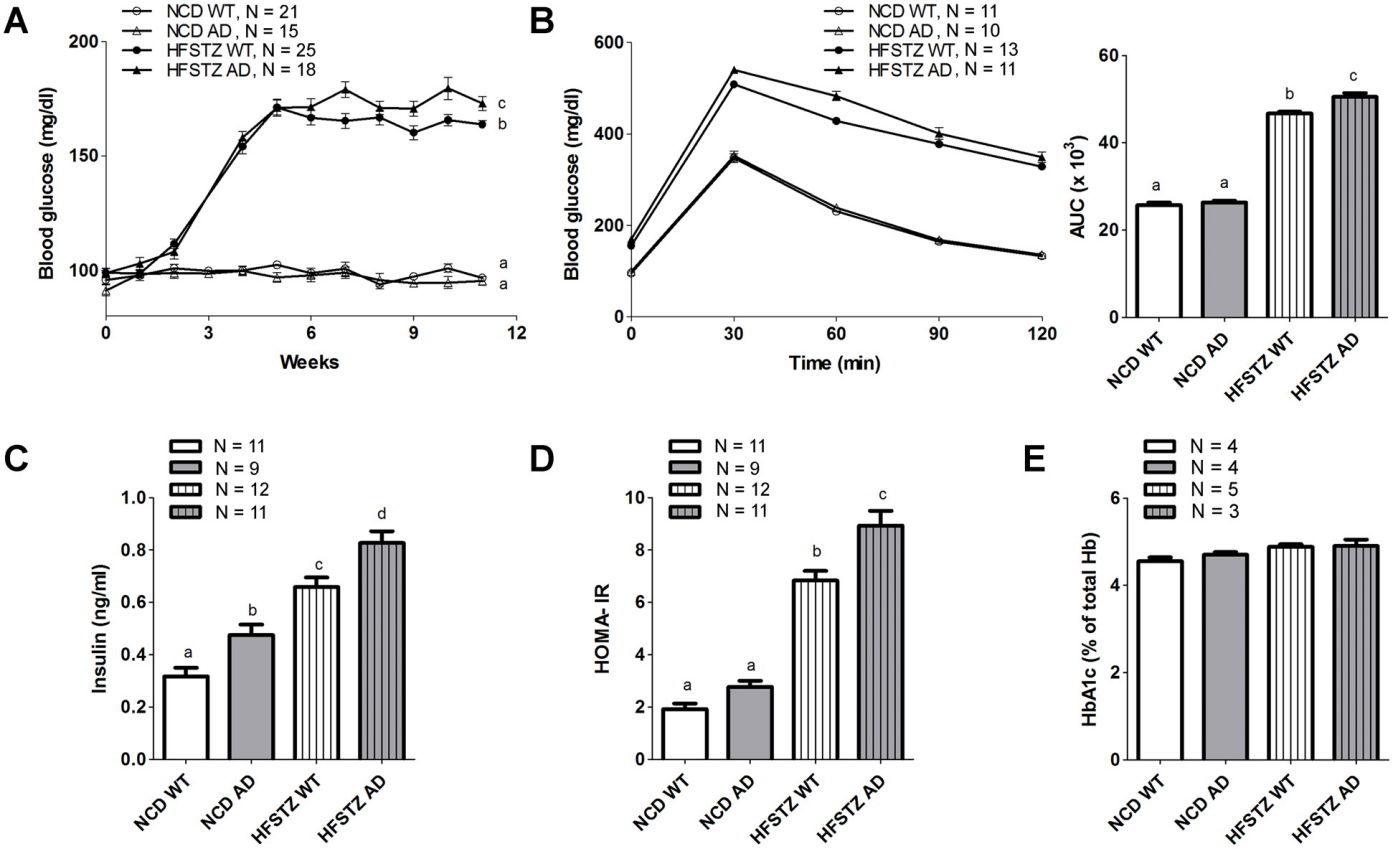
Impact of HFSTZ on glycemic control. WT or AD transgenic mice were treated with NCD or HFSTZ. (A) Fasting glucose levels by week. (B) Oral glucose tolerance testing was performed 6 weeks after dietary manipulation, and AUC values (right panel) were calculated. (C) Fasting insulin, (D) HOMA-IR, and (E) HbA1c% were determined at the end of the experiment. Bars represent the mean ± SEM. Experimental groups labeled with different letters are significantly different from each other (*p* < 0.05).

That employing a combination of a high fat diet and low-dose streptozotocin injections produced synergistic effects on metabolic disorders was first confirmed by two-way ANOVA analysis (S1 Table). HFSTZ resulted in significantly increased fasting blood glucose levels in both WT and APPswe/PS1dE9 transgenic (AD) mice 3 weeks after STZ injection (Fig 1A). In contrast, no differences in blood glucose were observed among NCD WT and AD mice. At the end of the experiment, the fasting blood glucose of HFSTZ AD mice was significantly higher than that of HFSTZ WT mice. When comparing glucose tolerance among the groups after 6 weeks, HFSTZ-induced glucose intolerance was worse in AD mice (higher area under the curve [AUC]) compared with that of WT mice (Fig 1B). For serum insulin concentration, we observed a stepwise increment of serum insulin levels in the following order: NCD WT, NCD AD, HFSTZ WT, and HFSTZ AD (Fig 1C). Although the mean HOMA-IR score of HFSTZ AD mice was consistently higher than that of HFSTZ WT mice, there was no difference in HOMA-IR between the NCD WT and NCD AD groups (Fig 1D). Interestingly, the glycated hemoglobin (HbA1c) percentage among the four groups was all approximately 4% and remained unchanged throughout the experiment (Fig 1E). Two-way ANOVA analysis indicated that the interaction of genetic type difference (APPswe/PS1dE9 *vs*. WT) and induced metabolic disorder (NCD *vs*. HFSTZ) was associated with increased fasting blood glucose (F _interaction (1, 75)_ = 6.641, *p* < 0.05) and glucose intolerance (F _interaction (1, 41)_ = 7.966, *p* < 0.01).

### HFSTZ AD mice became more obese and exhibited adipocyte hypertrophy and increased serum leptin concentrations

**Fig 2:**
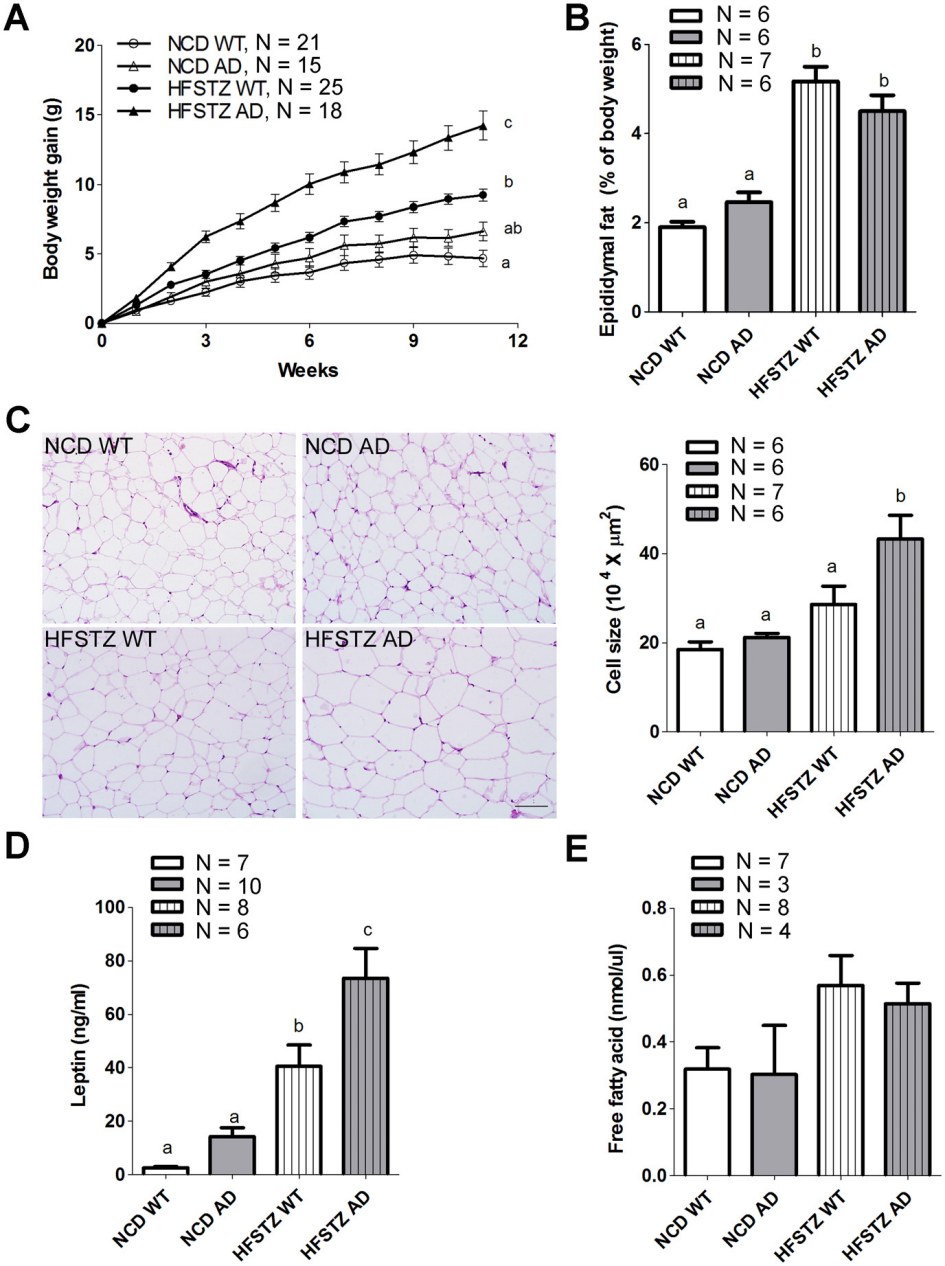
Characterization of obese conditions among each experimental group. WT or AD transgenic mice were treated with NCD or HFSTZ (A) Body weights were recorded by week. (B) Epididymal fat tissue was collected, fat mass estimation based on the percentage of epididymal fat of total body weight at after 11 weeks of dietary manipulations. (C) Representative histological microphotographs of HE-stained epididymal fat sections (scale bar, 50 μm). Adipocyte size was measured by ImageJ. (D) Serum leptin and (E) free fatty acid were quantified by ELISA after 11 weeks of dietary manipulation. Bars represent the mean ± SEM of at least three independent experiments. Experimental groups labeled with different letters are significantly different from each other (*p* < 0.05).

As shown in Fig 2A, the initial body weights of WT and AD mice were not different prior to the dietary manipulations. However, during the 11 weeks of the experiment, body weight gain in HFSTZ AD mice was significantly higher than that of HFSTZ WT mice. In contrast, there was no difference in body weight gain between NCD WT and NCD AD mice. The ratio of epididymal fat weight to body weight was significantly increased in HFSTZ WT and HFSTZ AD mice compared with NCD WT and AD mice at the end of experiment (Fig 2B). The mean size of adipocytes in HFSTZ AD mice was significantly enlarged compared with that of the other groups (Fig 2C).

Furthermore, the mean serum leptin concentration of HFSTZ AD mice was significant higher than that of HFSTZ WT mice (Fig 2D). There was no significant difference in serum free fatty acid among the groups (Fig 2E). Two-way ANOVA indicated that the interaction between genetic type and induced metabolic disorder lead to an increase of body weight gain (F _interaction (1, 75)_ = 4.835, *p* < 0.05) rather than fat mass.

**Fig 3:**
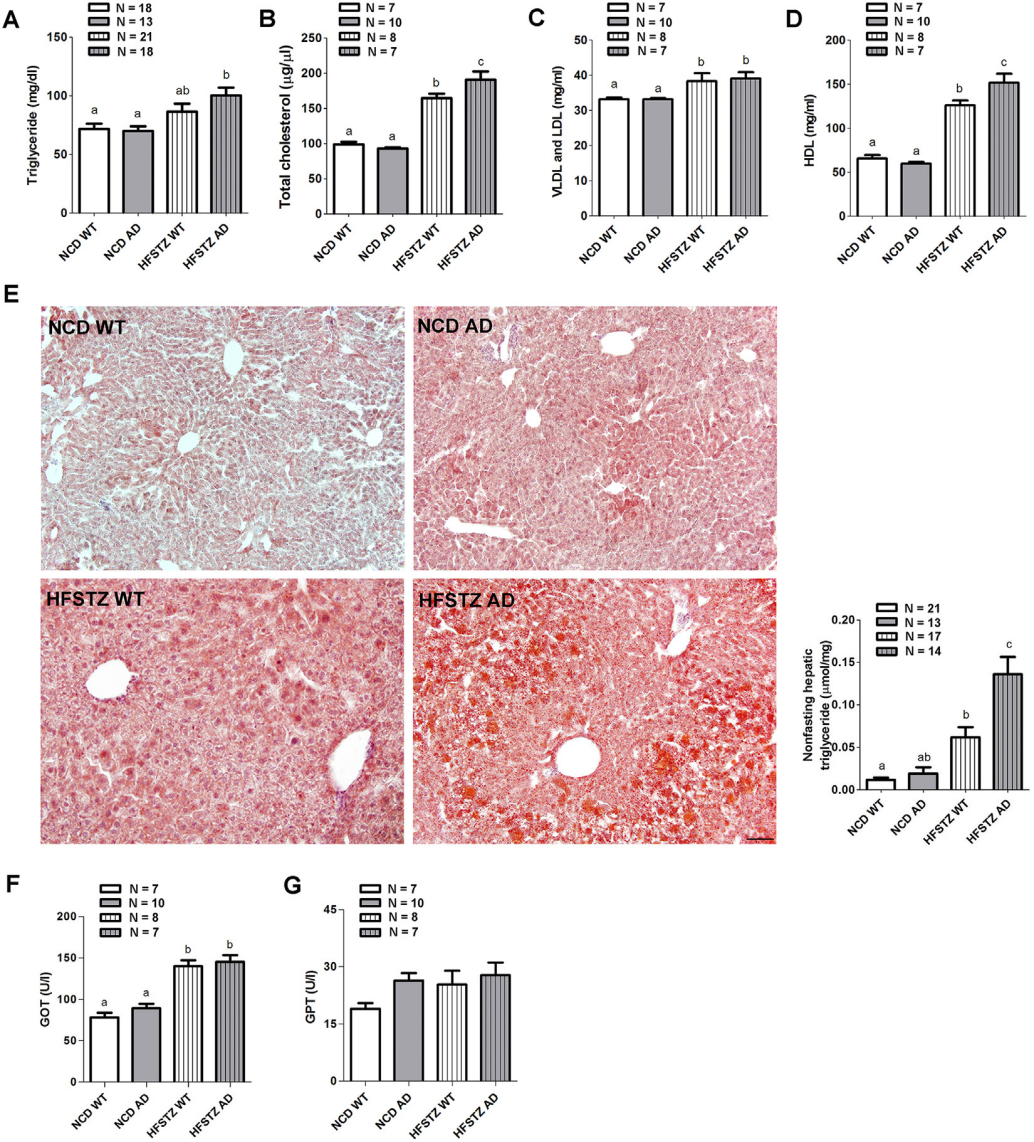
Analysis for serum lipid and hepatic steatosis. The levels of (A) serum TG, (B) total cholesterol, and cholesterol in (C) VLDL and LDL, as well as (D) HDL measured after 11-week dietary manipulations. (E) Representative histological microphotographs of liver sections stained with Oil Red O (scale bar, 50 μm) and hepatic TG contents of mice were quantified. (F) Serum GOT and (G) Serum GPT were also measured. Bars represent the mean ± SEM. Experimental groups labeled with different letters are significantly different from each other (*p* < 0.05).

Dyslipidemia and hepatic steatosis was exacerbated in HFSTZ AD mice.HFD consumption has been shown to lead to obesity and dyslipidemia [22]. Therefore, we continued to analyze serum lipid profiles at the end of our experiment. The serum level of TG significantly increased in HFSTZ AD mice, but not HFSTZ WT mice, as compared with NCD WT and AD mice (Fig 3A). In addition, the serum total cholesterol of HFSTZ-treated mice was significantly higher than that of NCD-fed mice, and HFSTZ AD mice had higher total cholesterol concentrations than HFSTZ WT mice (Fig 3B). HFSTZ treatment significantly increased the serum concentrations of VLDL and LDL as compared with the NCD groups; but there was no difference between AD and WT mice (Fig 3C). Interestingly, the pattern of changes in serum HDL was similar to that in the total cholesterol (Fig 3D). Two-way ANOVA revealed that AD genetic background interacted with HFSTZ to increase serum cholesterol levels (F _interaction (1, 28)_ = 6.598, *p* < 0.05). HFSTZ condition is known to cause dyslipidemia and increase hepatic TG levels in rodents [19]. Therefore, we performed histological and tissue TG assays to examine the extent of TG accumulation. There was severe hepatic TG staining in HFSTZ AD mice as shown by Oil Red O staining (Fig 3E, left panel). Consistently, liver TG content in HFSTZ AD mice was significantly higher than those measured in the other three groups (Fig 3E, right panel). In contrast, there were no differences in TG content in skeletal muscle among the four groups (data not shown). Two-way ANOVA analysis confirmed an interaction between APPswe/PS1dE9 genotype and HFSTZ on hepatic TG levels (F _interaction (1, 61)_ = 8.648, *p* < 0.05). In terms of liver function, serum GOT was significantly increased in HFSTZ AD and WT mice (Fig 3F). However, there was no significant difference in serum GPT among the groups (Fig 3G).

### Serum Aβ levels are correlated with metabolic indexes

**Fig 4:**
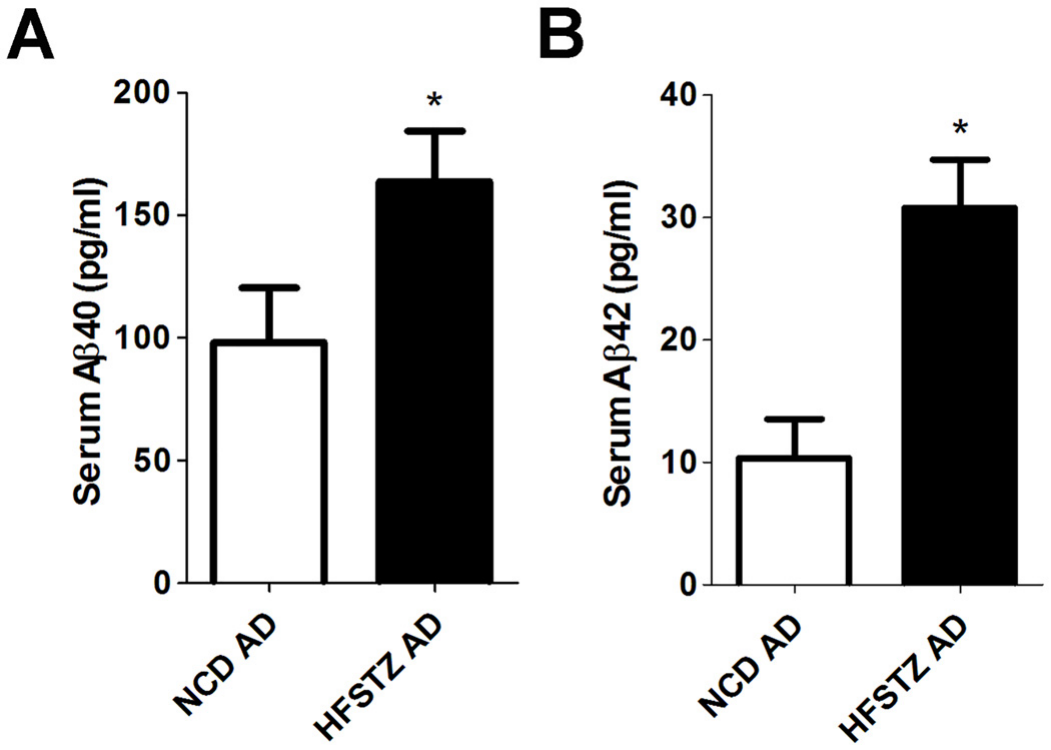
Serum Aβ40 and Aβ42 quantification. The levels of (A) Serum Aβ40 and (B) Serum Aβ42 of NCD and HFSTZ AD mice were measured after 11weeks of dietary manipulations by ELISA. Bars represent the mean ± SEM of at least three independent experiments. Significant differences (*p* < 0.001, unpaired t-tests) among the groups are labeled as ***.

Serum Aβ levels were higher in HFSTZ AD mice and were associated with the extent of hepatic steatosis, obesity, and elevated blood glucose. A previous study suggested that the serum level of Aβ in AD mice increases with elevated glucose intolerance and insulin resistance [16]. To assess whether hyperglycemia and hyperinsulinemia in HFSTZ AD mice correlated with serum Aβ, serum levels of Aβ40 (Fig 4A) and Aβ42 (Fig 4B) were measured. Both of them were significantly increased in AD mice after 11 weeks of HFSTZ treatment. Furthermore, the mean serum Aβ40 concentration in NCD AD mice was 10-fold higher than that of Aβ42. However, there was only a 5-fold difference between Aβ40 and Aβ42 in HFSTZ AD mice. The potential correlations between serum Aβ and weight gain, blood glucose, leptin, and hepatic TG from the data pool of AD mice under NCD and HFSTZ conditions were assessed by Pearson’s correlation analysis. As shown in Table 1, serum Aβ40 and Aβ42 levels were moderately correlated (R = 0.751). Both serum Aβ40 and Aβ42 levels were moderately correlated with body weight gain (R = 0.578 for Aβ40 and 0.727 for Aβ42) and blood glucose (R = 0.629 for Aβ40, 0.599 for Aβ42), and weakly correlated with leptin (R = 0.457 for Aβ40 and 0.445 for Aβ42). On the other hand, serum Aβ40 and Aβ42 levels were moderately and weakly correlated with hepatic TG, respectively (R = 0.511 for Aβ40 and 0.490 for Aβ42). However, serum Aβ40 and Aβ42 levels were not correlated with serum TG. Consistently, weight gain, blood glucose, leptin, and epididymal fat weight (% of body weight) were more correlated with hepatic TG than serum TG.

**Table 1:** Pearson correlation analysis of serum Aβ and metabolic markers of APP/PS1 transgenic mice after 11-week dietary manipulations.

		Aβ40	Aβ42	BW gain	Blood glucose	leptin	hepatic TG	fat(% of BW)	serum TG
Aβ40	R	1	0.75**	0.58**	0.63**	0.46*	0.51*	0.30	0.10
	Sig		<0.001	<0.001	<0.001	0.022	0.011	0.143	0.681
	N		33	33	30	25	24	25	18
Aβ42	R		1	0.73**	0.60**	0.45*	0.49*	0.47*	-0.36
	Sig			<0.001	<0.001	0.026	0.015	0.019	0.125
	N			33	30	25	24	25	18
BW gain	R			1	0.56**	0.84**	0.82**	0.71**	0.13
	Sig				<0.001	<0.001	<0.001	<0.001	0.481
	N				43	26	24	26	30
Blood glucose	R				1	0.45**	0.72**	0.68**	0.40*
	Sig					0.031	<0.001	<0.001	0.03
	N					23	21	23	30
leptin	R					1	0.71**	0.63**	0.77**
	Sig						<0.001	0.001	0.009
	N						24	26	10
hepatic TG	R						1	0.72**	0.67*
	Sig							<0.001	0.036
	N							24	10
fat (% of BW)	R							1	0.80**
	Sig								0.005
	N								10
serum TG	R								1
	Sig								
	N								

**P< 0.01 level (2-tailed) and

* *P*< 0.05 level (2-tailed).

BW: body weight; fat (% of BW): epididymal fat weight (% of body weight); R: regression coefficient.

## Discussion

Previous studies have shown that diabetic and/or hyperlipidemic conditions could have significant impacts on CNS pathology in aged AD mice. This study is focused on the interaction of APPswe/PS1dE9 genetic background and metabolic stresses in mice at the prodromal stage of AD. Our data suggests that HFSTZ-induced metabolic stresses are aggravated in young adult AD mice compared with WT mice. Furthermore, hepatic steatosis was exacerbated in HFSTZ-treated AD mice, and peripheral Aβ levels were positively correlated with HFSTZ-induced metabolic changes, including hepatic steatosis. Ours is the first study to reveal the synergistic interaction between APPswe/PS1dE9 genetic background and HFSTZ in exacerbating a broad spectrum of metabolic stresses including hepatic steatosis at the early stage of AD pathogenesis.

High fat diet or genetic manipulations of leptin signaling such as the mutation of leptin and leptin receptor increase AD-like pathology at the advanced stage in AD mouse models [11, 12, 15, 23]. In contrast to other models of metabolic disorders, HFSTZ utilized in this study induces mild metabolic disturbance. For example, the fasting blood glucose levels in HFSTZ WT mice fluctuate around 150 mg/dL; however, levels in db/db mice usually exceed 400 mg/dL at the same age. The HbA1c percent in HFSTZ-treated mice also appears to be within the normal range [12]. These data suggest that the HFSTZ treatments in general introduce a mild metabolic disturbance. Furthermore, the mild but significant alterations of liver functions could lead to the increased peripheral Aβ in HFSTZ AD mice. The statistical analyses from our results reveal an important message: the reciprocal interactions among hepatic steatosis, metabolic stresses, and elevated serum Aβ exist in mice bearing both mutant APPswe/PS1dE9 transgenes and diabesity.

It has been shown that the liver is the major organ for the clearance of plasma Aβ [24, 25]. The observation of elevated peripheral Aβ in HFSTZ-treated AD mice might indicate a vicious cycle between hepatic steatosis and Aβ clearance to exacerbate existing metabolic stresses. The accumulation of peripheral Aβ in HFSTZ-treated AD mice found in our study can be at least partly attributed to abnormal liver function. Thus, mild but significant alterations of liver function, especially pertaining to Aβ degradation and/or excretion, are likely to take place. Subsequently, Aβ species in the blood are incorporated into HDL for delivery to the liver. Thus, hypercholesterolemia might be associated with elevated serum Aβ. Indeed, higher serum Aβ is reported among cognitively normal non-statin users in AD family history-enriched cohorts. The same study also found that the levels of serum Aβ and HDL were positively associated [26]. Our study further demonstrates that HFSTZ AD mice at the early stage of AD were hypercholesterolemic and had higher levels of both total and HDL cholesterol as compared with the other three groups. These findings suggest that peripheral HDL may play an important role in peripheral Aβ homeostasis as well as in the development of AD. Regulation of Aβ drain out from CNS is linked to peripheral Aβ level. And Aβ degradation by the liver appears to play important role for such dynamic regulation [27]. Therefore, we may expect that elevated peripheral Aβ in HFSTZ AD mice would exacerbate Aβ burden in CNS at later stage.

In conclusion, we present evidence that APPswe/PS1dE9 genetic background interacts with the development of peripheral metabolic stresses induced by HFSTZ at the early stage of AD. The consequential effects are exacerbated dysglycemic control, obesity, and hepatic steatosis. We speculate that mild but significant alteration of liver function coupled with worsened metabolic stresses in HFSTZ AD mice might contribute to the elevated levels of serum Aβ. Our findings suggest that treating hepatic steatosis could be of clinical importance, especially in the prodromal stage of AD.

## Supporting Information

S1 Table The interaction of diets and STZ on metabolic parameters of WT mice. (DOCX)

S1 Checklist Supporting information NC3Rs ARRIVE Guidelines checklist 2014. (DOCX)
