# Supplementary material for: Obesity and Hepatic Steatosis Are Associated with Elevated Serum Amyloid Beta in Metabolically Stressed APPswe/PS1dE9 Mice
Source: PLoS One. 2015 Aug 5;10(8):e0134531. doi: 10.1371/journal.pone.0134531 (PMC4526466; doi:10.1371/journal.pone.0134531)
Supplement: S1 Table — (DOCX) [file pone.0134531.s001.docx]

## Table S1. The Interaction of Diets and STZ on Metabolic Parameters of WT Mice.

|  | Interaction,  F_interaction_  (P value) | Simple main effect,  F_group_  (P value) | | | |
| --- | --- | --- | --- | --- | --- |
|  |  | NCD | HFD | Vehicle | STZ |
| Body weight gain | 29.831  (<0.001) | 0.005  (0.944) | 69.564 (<0.001) | 133.525 (<0.001) | 34.340  (<0.001) |
|  |  |  |  |  |  |
| Blood glucose | 57.674  (<0.001) | 41.611 (<0.001) | 269.681 (<0.001) | 96.461 (<0.001) | 249.505  (<0.001) |
|  |  |  |  |  |  |
| AUC of OGTT | 12.718  (0.001) | 125.924 (<0.001) | 275.029 (<0.001) | 349.309 (<0.001) | 777.127  (<0.001) |
|  |  |  |  |  |  |
| Insulin | 37.152  (<0.001) | 46.204 (<0.001) | 7.78  (0.011) | 68.116 (<0.001) | 0.553  (0.466) |
|  |  |  |  |  |  |
| HOMA-IR | 5.986  (0.019) | 81.732 (<0.001) | 27.996 (<0.001) | 50.807 (<0.001) | 50.807  (<0.001) |

AUC of OGTT: area under curve of oral glucose tolerance test.
