# Supplementary material for: Obesity and Hepatic Steatosis Are Associated with Elevated Serum Amyloid Beta in Metabolically Stressed APPswe/PS1dE9 Mice
Source: PLoS One. 2015 Aug 5;10(8):e0134531. doi: 10.1371/journal.pone.0134531 (PMC4526466; doi:10.1371/journal.pone.0134531)

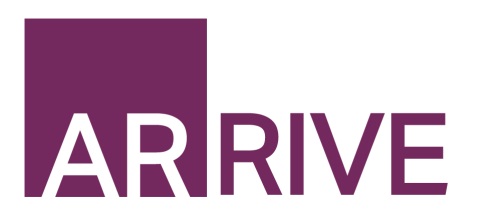


The ARRIVE Guidelines Checklist

Animal Research: Reporting In Vivo Experiments

Carol Kilkenny^1^, William J Browne^2^, Innes C Cuthill^3^, Michael Emerson^4^ and Douglas G Altman^5^

*^1^The National Centre for the Replacement, Refinement and Reduction of Animals in Research, London, UK, ^2^School of Veterinary Science, University of Bristol, Bristol, UK, ^3^School of Biological Sciences, University of Bristol, Bristol, UK, ^4^National Heart and Lung Institute, Imperial College London, UK, ^5^Centre for Statistics in Medicine, University of Oxford, Oxford, UK.*

|  | | ITEM | RECOMMENDATION | Section/ Paragraph |
| --- | --- | --- | --- | --- |
| 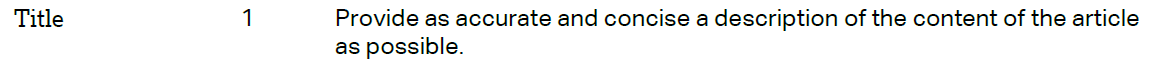 | | | page1/1 |  |
| 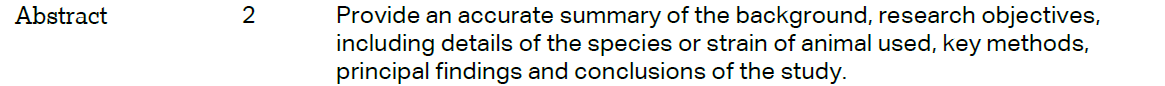 | | | p2/1 |  |
| INTRODUCTION | | |  |  |
| 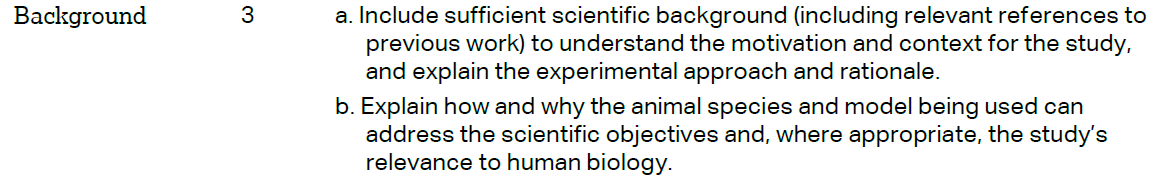 | | | p2 |  |
| 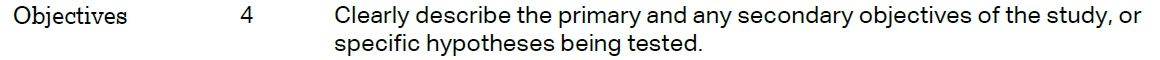 | | | p4/3 |  |
| METHODS | | |  |  |
| 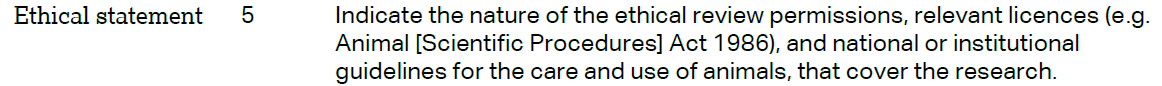 | | | p5/1 |  |
| 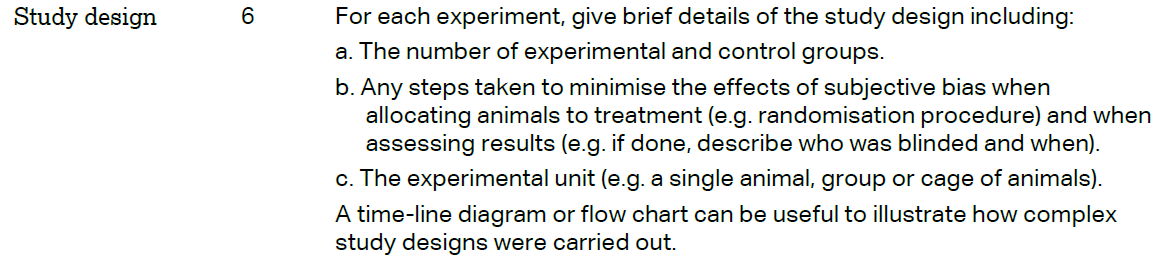 | | | p5/2 |  |
| 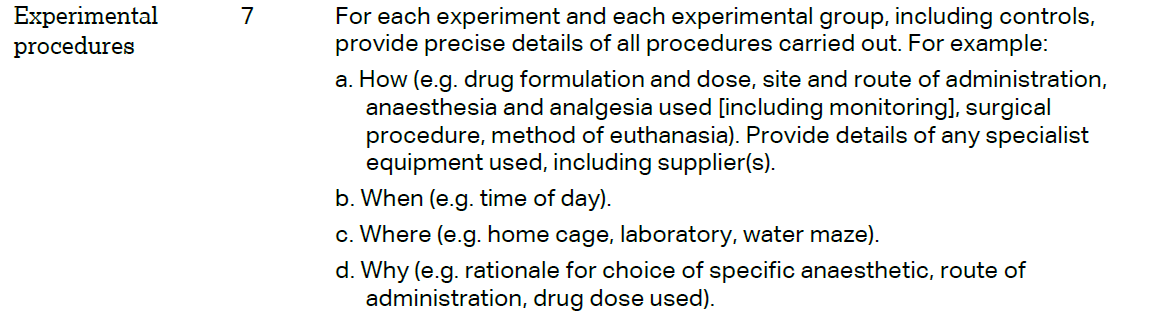 | | | p6/1-P8 |  |
| 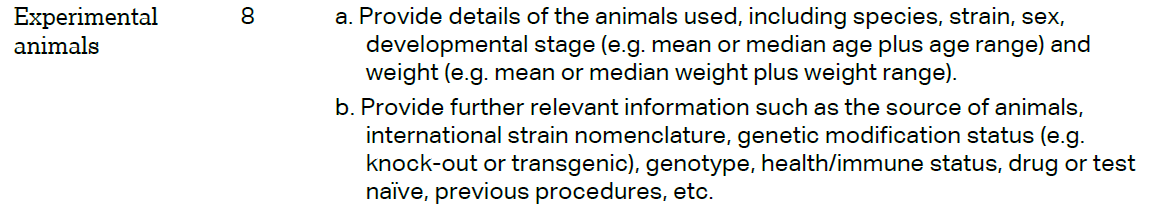 | | | p5/1 |  |

The ARRIVE guidelines. Originally published in *PLoS Biology*, June 2010^1^

| 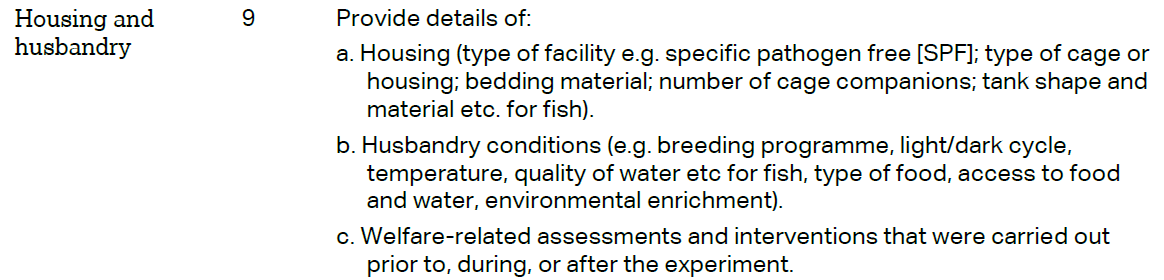 | p5/1 | |
| --- | --- | --- |
| 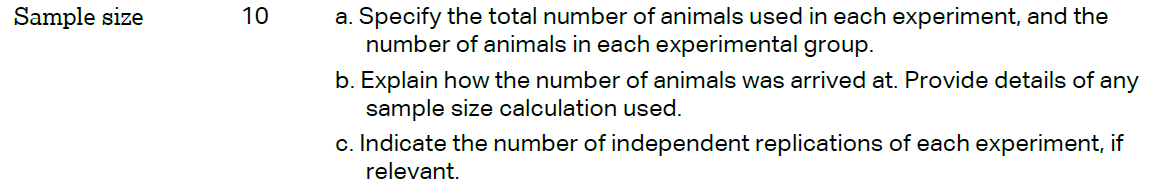 | p5/1  figures  p8/1 | |
| 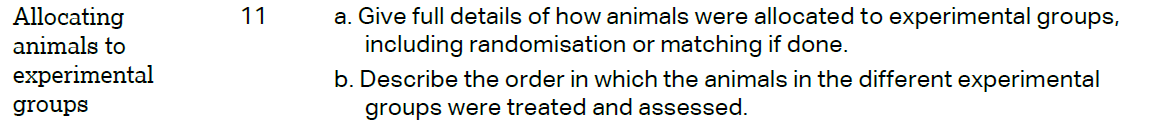 | p5/2 | |
| 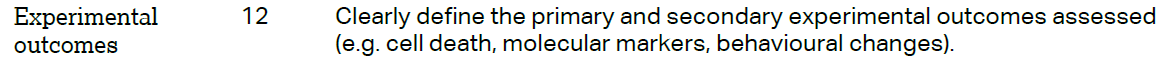 | p6/2-p7 | |
| 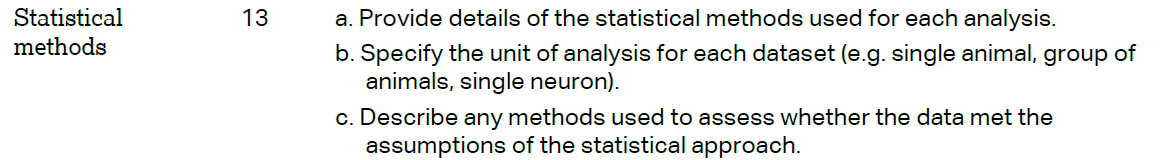 | p7/4 | |
| RESULTS |  | |
| 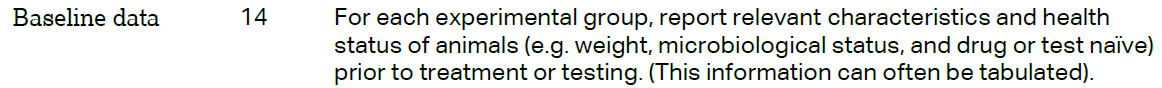 | p8/2 | |
| 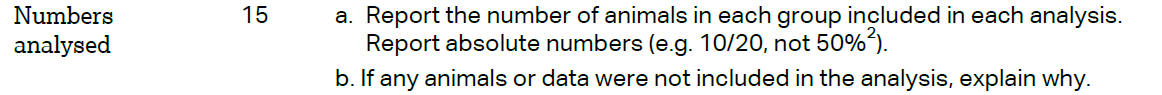 | p5/3, p8/2 | |
| 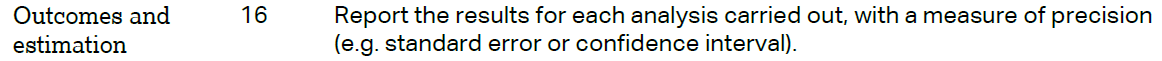 | figures and p12 | |
| 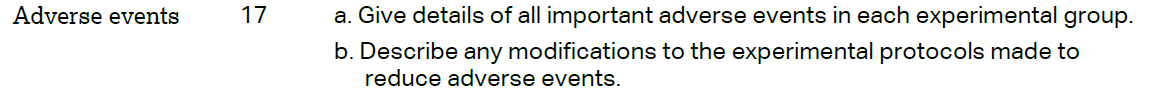 | No | |
| DISCUSSION |  | |
| 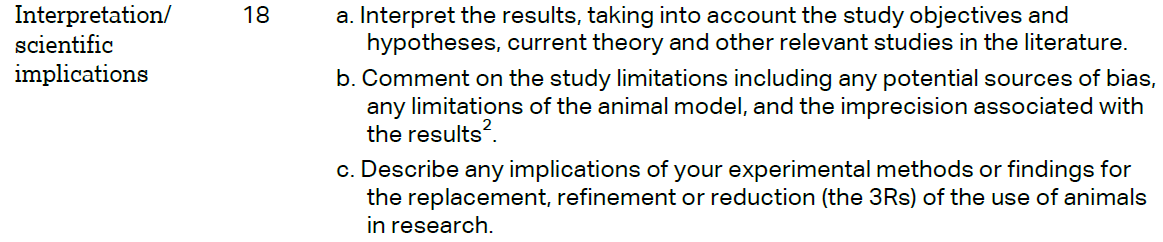 | p13-p15 | |
| 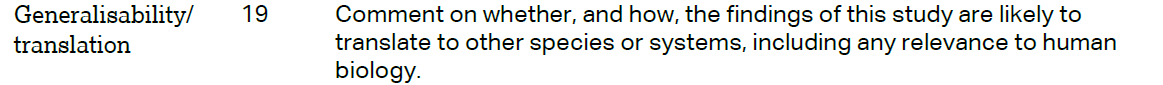 | p15/3 | |
| 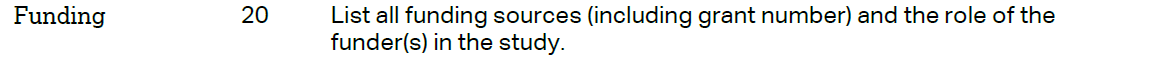 | | submittion step 5 |


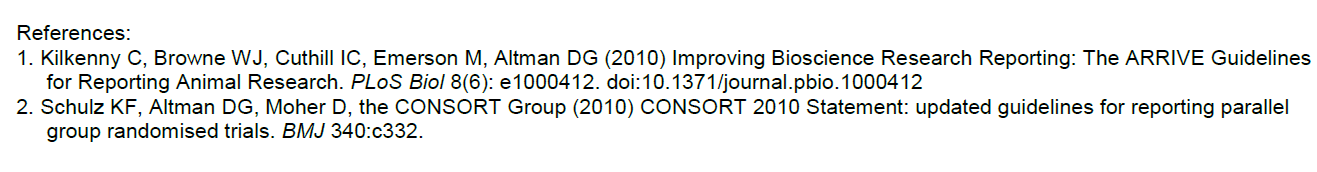

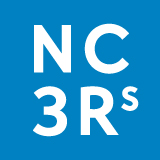

Supplement: S1 Checklist — (DOCX) [file pone.0134531.s002.docx]
